# Clinicopathologic significance of heat shock protein 60 as a survival predictor in breast carcinoma

**DOI:** 10.3389/fonc.2024.1415762

**Published:** 2024-08-19

**Authors:** Qing Wang, Shengzhou Chen, Zhihong Wu, Jungang Ni

**Affiliations:** Department of Thyroid and Breast Oncology, Yiwu Central Hospital, Yiwu, Zhejiang, China

**Keywords:** HSP60, prognosis, overall survival, progression-free survival, breast carcinoma

## Abstract

**Background:**

While Heat Shock Protein 60 (HSP60) has been linked to human tumor, its clinic significance specifically in breast carcinoma is unclear. This investigation aims to retrospectively evaluate how HSP60 protein levels relate to survival outcomes among patients diagnosed with breast carcinoma.

**Methods:**

Evaluation of 206 patients diagnosed with breast carcinoma and receiving treatment from January 2012 to April 2018, carried out retrospectively. The protein level of HSP60 in breast carcinoma determined by immunohistochemical.

**Results:**

The study provided evidence of a distinct upregulation of HSP60 expression in breast carcinoma tumor samples in contrast to adjacent normal tissue samples. Additionally, heightened HSP60 expression was linked to advanced T stage (P = 0.046), N stage (P = 0.034), tumor metastasis (P = 0.016), pathological grading (P = 0.012), and adjuvant therapy utilization (P = 0.004). Moreover, elevated levels of HSP60 proteins exhibited a significant inverse correlation with overall survival (OS) [hazard ratio (HR) 1.598, P = 0.018] and progression-free survival (PFS) (HR 1.600, P = 0.017) among breast carcinoma patients in univariate analyses. The results of multivariate analyses highlighted HSP60 may serve as an independent predictor for both OS and PFS in breast carcinoma patients (HR 1.525, P = 0.034; HR 1.528, P = 0.033, respectively).

**Conclusion:**

The involvement of HSP60 in breast carcinoma progression suggests its potential clinical relevance in treatment target validation and prognostic assessment of the disease.

## Introduction

Among the female population, breast carcinoma is one of the more prevalent types of malignant tumors. According to research, estimates suggest that after 2040, the global annual incidence of new breast carcinoma cases will exceed 3 million, and more than one-third of breast carcinoma patients will die every year (1, 2). Based on reports, the five-year overall survival (OS) rate for breast carcinoma in Asia is 66-69% (3). In the past 40 years, the incidence rate of breast carcinoma has shown a significant upward trend (4, 5).

The evolution of cancer treatment paradigms has led to the development of targeted therapies, resulting in improved rates of cancer survival (6). The focus of targeted therapeutics is on specific factors involved in cancer advancement. As a result, their effectiveness within a particular cancer type may be limited to subsets of patients characterized by the expression of the targeted factor. Finally, the revelation of fresh markers for carcinoma subgroups represents the foundational stage in the advancement of targeted therapies. Accordingly, there is a critical need to identify new biomarkers for early detection, prognostic assessment, and targeted therapy of breast carcinoma.

Heat shock protein 60 (HSP60), produced from the heat shock protein family D member 1 (HSPD1) gene, is a mitochondrial protein encoded by the nucleus, acting as a molecular chaperone within the matrix compartment (7). Additionally, it inhibits the abnormal folding or clustering of proteins, assists in the intracellular trafficking of proteins to organelles, and breaks down unfolded proteins (8). HSP60 is also implicated wide spectrum of biological processes, including proliferation (9), cell cycle regulation (10), oxidative stress (11), and apoptosis (12), within diverse cancer types such as gynecological and digestive system tumors (13–15). Despite this, the significance of HSP60 in cancer prognosis continues to be disputed. For example, in oral squamous cell cancer and non-small cell lung cancer, heightened levels of HSP60 expression are linked to poorer prognosis, leading to reduced OS (16, 17). However, in ovarian cancer patients, prognosis appears favorable due to the significant promotion of ovarian cancer cell proliferation and migration upon HSP60 knockdown (18).

The optimal method for interpreting and assessing HSP60 in clinical practice remains a matter of debate among pathologists. Moreover, the importance of HSP60 expression and its clinical implications in breast carcinoma remain inadequately investigated. This study involved performing immunohistochemical analysis to assess the link between HSP60 protein levels and the prognosis of breast carcinoma.

## Methods

### Patients

During the timeframe of January 2012 to April 2018, our hospital gathered cancerous tissue samples (n = 237) and corresponding adjacent normal tissue samples (n = 42) from patients undergoing breast carcinoma surgery. Immunohistochemistry (IHC) staining analysis were conducted on tumor tissues from breast carcinoma patients to assess their survival duration based on HSP60 protein levels.

The medical data of all patients were carefully evaluated and sanctioned for use as authorized by the Medical Ethics Committee of Yiwu Central Hospital. The subsequent selection criterion was used (1): Subjects from the Chinese population who underwent radical surgery for primary breast carcinoma and were diagnosed with breast carcinoma through tissue biopsy or imaging examination (2); Patients with sufficient and valid clinical data. The following is the definition of exclusion criteria: (1) individuals with coexisting breast conditions, autoimmune disorders, or current use of anticoagulant medications (n =7); (2) Patients suffering from other serious illnesses such as cardiovascular diseases, liver diseases, or other cancers (n = 6); (3) Exclude patients with other inflammatory diseases. (n = 5); (4) Patients who have received specific treatment within the past 3 months should be excluded (n = 7); and (5) Individuals with inadequate or unreliable medical clinical data (n = 6). Initially, 237 samples were included in the study. After screening, 31 samples were excluded for not meeting the requirements, and ultimately, 206 consecutive patients diagnosed with breast carcinoma were retrospectively enrolled in the present study.

The clinical information covered in cancer patients’ medical records includes, but is not limited to: age, cancer (T) stage, node (N) stage, metastasis stutas, ER, PR, HER2, treatment modalities (such as surgery, chemotherapy, radiotherapy, targeted therapy, etc), treatment course and response, adverse events, and survival status. In all samples, the diagnosis of breast carcinoma was confirmed histopathologically by two different pathologists. All patients were pathologically confirmed to have breast carcinoma and staged according to the UICC TNM 8th edition.

Patients diagnosed with breast carcinoma were typically monitored through phone calls or postoperative follow-up. OS is typically defined as the duration from the initiation of treatment or surgery in a patient until death or the last follow-up. (October 2, 2023) or death due to any cause. The definition of progression-free survival (PFS) involved measuring the duration from the initiation of surgical therapy to the onset of the first disease progression, indicated by any progression (any advancement in tumor clinical staging and pathological grading). The follow-up duration reached its midpoint at 67 months, representing a range of 3 to 99 months.

### Immunohistochemistry

The staining for HSP60 via IHC was implemented following the manufacturer’s instructions. Paraffin-embedded mammary tissue and breast carcinoma tissue microarray slides underwent IHC staining. A gradient concentration of ethanol hydrate was applied after dewaxing 4 μm paraffin sections three times in xylene.

The slides underwent a 1-hour incubation with anti-HSP60 antibody at 1:100 dilutions (ab190828; Abcam, Cambridge, UK) overnight at 4°C following recovery of the antigen and blocking of endogenous peroxidase activity. Presence of brown chromophores in the nucleus and cytoplasm of target cells indicated a positive immunoreaction. Following staining optimization through scoring negative and positive controls, optical microscopy at 400× magnification was used to examine the slide.

### Evaluation of the IHC results

Irrespective of the staining intensity, HSP60-positive cells were evaluated, followed by the assessment of intracytoplasmic staining of HSP60. Assessment of the HSP60 score followed the methodology outlined by Li et al. (19).

Recorded by two observers (SC and ZW), positive tumor cell percentages were allocated as 0 points (<5%), 1 point (5–10%), 2 points (11–50%), 3 points (>50%). Each sample achieved a total score between 0 and 3 considering HSP60 protein levels.

In cases where multiple observers provided identical scores, such scores were accepted as the definitive assessment for each specimen. The intensity of HSP60 staining determined the score, the intensity (0-negative, 1-weak, 2-moderate and 3-strong). The total score, ranging between 0 and 3, was derived by summing the intensity score with the stained area percentage product. Designation of final scores as either low (≤1) or high (≥2) occurred subsequent to computation.

### Statistical analysis

Comparison of HSP60 IHC staining in cancerous tissue samples and paracancerous mammary tissue counterparts was analyzed utilizing GraphPad Prism 10 software, with either the t-test or chi-square test employed.

During deep sectioning of HSP60 IHC, the tumor component was lost in four out of the 46 tumor tissues, rendering the assessment of HSP60 expression in these tumors impossible. Accordingly, these four carcinoma samples were left out of the analyses concerning carcinoma constituent expression. 42 breast carcinoma samples and their corresponding adjacent normal tissues were incorporated in the subsequent assessment. SPSS 26.0 software (SPSS Inc., Chicago, IL, USA) was employed for statistical analyses, with significance set at P < 0.05. The link between HSP60 protein levels and clinical parameters was evaluated via Fisher’s exact test. By definition, OS denoted death resulting from the tumor, and PFS was precisely characterized as the duration from the initial surgical therapy to the first indication of disease progression. Both univariable and multivariable assessments were utilized to investigate the comparison of OS and PFS between elevated HSP60 levels and reduced HSP60 levels.

HSP60 expression, tumor T stage, N stage, and Pathologic stage were divided into distinct categories: high compared to low levels of HSP60, early stage (T0, Tis, T1) compared to late stage (T2-4), N stage (N0) compared to N stage (N1-3), and M stage (M0) compared to M stage (M1).

## Results

### HSP60 protein expression is heightened in individuals diagnosed with breast carcinoma

**Figure 1:**
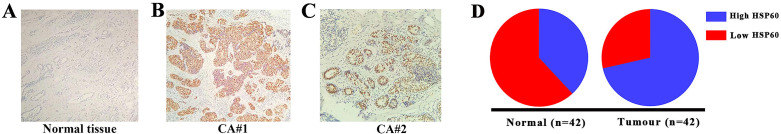
The determination of HSP60 protein levels in breast carcinoma tissues was conducted via immunohistochemical staining (original magnification, 200×). **(A–C)** Illustrates representative HSP60 protein levels in tumor and normal tissues, where positive expression was observed in the nucleus. **(D)** It showed that the high level of HSP60 expression in breast carcinoma was higher than in adjacent normal tissues.

In 42 pairs of breast carcinoma and their corresponding adjacent samples, HSP60 protein expression was determined utilizing the IHC staining technique. Representative photomicrographs illustrating HSP60 immunohistochemical staining are depicted in **Figures 1A–C**. According to the scatter dot plot, the mean immunoreactivity score of HSP60 protein in 42 breast carcinoma specimens was notably elevated compared to their corresponding 42 adjacent tissue specimens (carcinoma samples vs. non-cancerous tissue samples: 30/42 vs. 16/42, p < 0.01) (**Figure 1D**; **Supplementary Table S1**).

### Analysis of clinical parameters according to HSP60 immunoexpression level

**Table 1** outlines the connection between HSP60 protein levels and clinical-pathological parameters in breast carcinoma. A notable connection was observed between high HSP60 expression and aggressive characteristics such as high T stage (P = 0.046), N stage (P = 0.034), tumor metastasis (P = 0.016), pathological grading (P = 0.012), and adjuvant therapy (P = 0.004), as indicated by the results. But there was no statistical significance between HSP60 protein level and age, pathologic stage, ER, PR, HER2 status, and neoadjuvant chemotherapy (All P > 0.05).

**Table 1:** The relationship between the high and low expression of HSP60 and different clinical indicators in patients with 206 breast carcinoma.

Parameter	Overall	High expression of HSP60	Low expression of HSP60	P value
n	206	128	78	
Age, n (%)				
< = 60	137	86	51	0.790
> 60	69	42	27	
T stage, n (%)				
T0/Tis/T1	53	39	14	0.046
T2/T3/T4	153	89	64	
N stage, n (%)				
N0	104	72	32	0.034
N1-3	102	56	46	
M stage, n (%)				
M0	170	112	58	0.016
M1	36	16	20	
Pathologic stage, n (%)				
Stage I- II	109	59	50	0.012
Stage III- IV	97	69	28	
ER status, n (%)				
Negative	61	42	19	0.197
Positive	145	86	59	
PR status, n (%)				
Negative	79	54	25	0.147
Positive	127	74	53	
HER2 status, n (%)				
Negative	108	70	38	0.405
Positive	98	58	40	
Adjuvant therapy				
No	145	81	64	0.004
Yes	61	47	14	
Neoadjuvant chemotherapy				
No	116	74	42	0.578
Yes	90	54	36	

ER, estrogen receptor; HER2, human epidermal growth factor receptor; HSP60, Heat Shock Protein 60; PR, progesterone receptor. Bold values indicate that p < 0.05.

### Survival analysis

**Figure 2:**
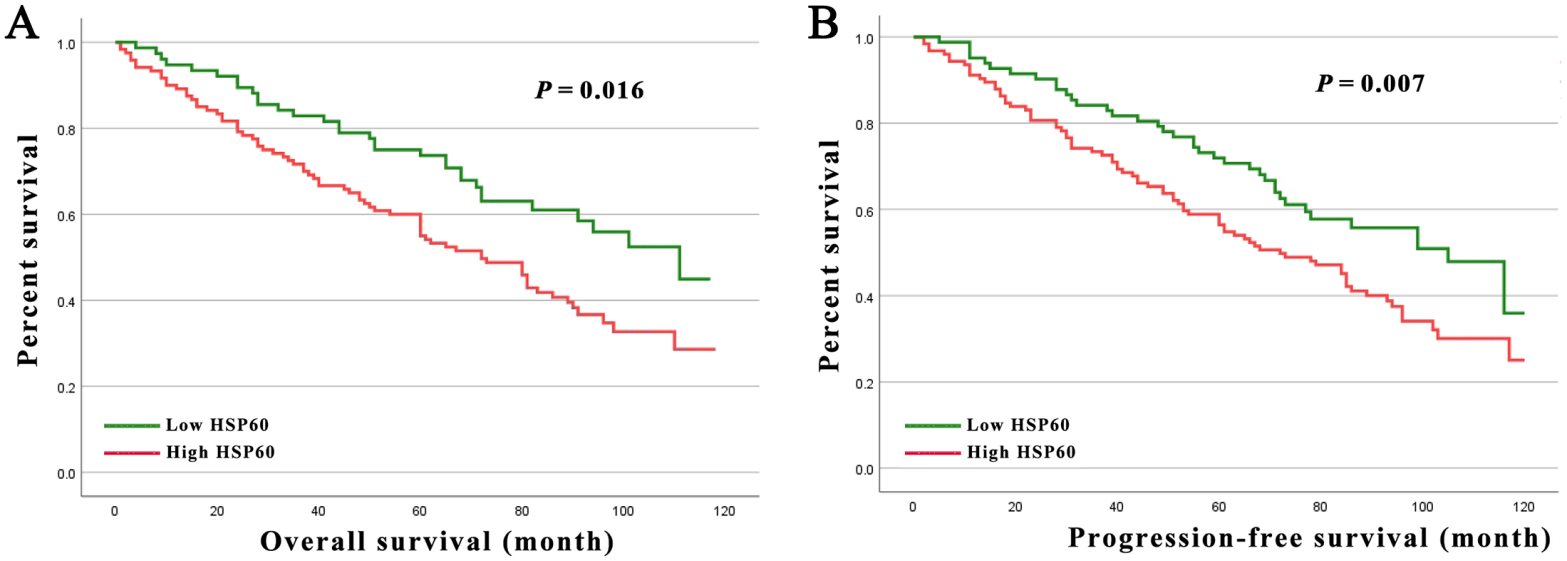
**(A)** Kaplan-Meier curves for the 5-year OS rate of patients with breast carcinoma stratified by HSP60 protein level in 206 breast carcinoma patients. **(B)** Kaplan-Meier curves for the 5-year PFS rate of patients with breast carcinoma stratified by HSP60 protein level in 206 breast carcinoma patients.

Higher HSP60 expression in Kaplan-Meier analyses was linked to shorter OS and PFS for breast carcinoma patients (**Figures 2A, B**, all P < 0.05). Moreover, this was corroborated by the projected cumulative 5-y OS (high exhibited 58.3% versus low with 78.1%) and 5-y PFS (high exhibited 55.4% versus low with 75.3%) rates for breast carcinoma patients presenting varying levels of HSP60 expression.

The risk of death was examined through univariate analysis, indicating that T stage (P = 0.006 and P = 0.005), N stage (P = 0.014 and P = 0.011), tumor metastasis (P = 0.001 and P = 0.001), pathologic stage (P = 0.026 and P = 0.022), and HSP60 expression (P = 0.018 and P = 0.017) were significantly connected with both OS and PFS. Adjuvant therapy (P = 0.049) was additionally linked to PFS (**Tables 2**, **3**). After adjusting for confounding variables, multivariate analysis showed that T stage, N stage, tumor metastasis, and HSP60 correlated obviously with OS (P = 0.002, P = 0.022, P < 0.001, P = 0.034, respectively) and PFS (P = 0.002, P = 0.016, P < 0.001, P = 0.033, respectively).

**Table 2:** Univariate and multivariable analysis of factors potentially predictive of overall survival.

Characteristics	Univariate			Multivariate		
	Hazard Ratio	95%CI	P-value	Hazard Ratio	95%CI	P-value
Age, years						
≥65vs<65	0.961	0.667-1.385	0.831	0.902	0.620-1.312	0.590
T stage, n (%)						
T2/T3/T4 vs T0/Tis/T1	1.692	1.162-2.465	0.006	1.823	1.245-2.669	0.002
N stage, n (%)						
N1-3 vs N0	1.593	1.097-2.313	0.014	1.567	1.067-2.302	0.022
M stage, n (%)						
M1 vs M0	1.819	1.262-2.623	0.001	2.104	1.446-3.062	<0.001
Pathologic stage, n (%)						
Stage III- IV vs Stage I- II	1.546	1.054-2.267	0.026	1.321	0.893-1.954	0.163
ER status, n (%)						
Positive vs Negative	0.855	0.592-1.233	0.401	0.839	0.580-1.212	0.349
PR status, n (%)						
Positive vs Negative	0.940	0.651-1.359	0.744	1.025	0.700-1.500	0.899
HER2 status, n (%)						
Positive vs Negative	1.005	0.694-1.455	0.979	1.072	0.734-1.565	0.719
Adjuvant therapy						
No vs Yes	1.453	0.989-2.135	0.057	1.454	0.986-2.144	0.059
Neoadjuvant chemotherapy						
No vs Yes	1.064	0.739-1.531	0.739	1.145	0.790-1.661	0.475
HSP60						
High vs Low	1.598	1.084-2.355	0.018	1.525	1.032-2.253	0.034

ER, estrogen receptor; HER2, human epidermal growth factor receptor; HSP60, Heat Shock Protein 60; PR, progesterone receptor. Bold values indicate that p < 0.05.

**Table 3:** Univariate and multivariable analysis of factors potentially predictive of progression-free survival.

Characteristics	Univariate			Multivariate		
	Hazard Ratio	95%CI	P-value	Hazard Ratio	95%CI	P-value
Age, years						
≥65vs<65	0.977	0.678-1.408	0.900	0.909	0.625-1.323	0.618
T stage, n (%)						
T2/T3/T4 vs T0/Tis/T1	1.719	1.181-2.503	0.005	1.843	1.260-2.696	0.002
N stage, n (%)						
N1-3 vs N0	1.620	1.116-2.353	0.011	1.607	1.094-2.362	0.016
M stage, n (%)						
M1 vs M0	1.813	1.258-2.613	0.001	2.129	1.462-3.101	<0.001
Pathologic stage, n (%)						
Stage III- IV vs Stage I- II	1.565	1.067-2.595	0.022	1.331	0.900-1.968	0.152
ER status, n (%)						
Positive vs Negative	0.849	0.589-1.224	0.380	0.835	0.578-1.208	0.338
PR status, n (%)						
Positive vs Negative	0.923	0.639-1.334	0.670	1.002	0.685-1.465	0.991
HER2 status, n (%)						
Positive vs Negative	1.013	0.700-1.467	0.946	1.079	0.739-1.575	0.694
Adjuvant therapy						
No vs Yes	1.472	1.002-2.164	0.049	1.461	0.991-2.154	0.055
Neoadjuvant chemotherapy						
No vs Yes	1.057	0.734-1.520	0.766	1.146	0.790-1.663	0.472
HSP60						
High vs Low	1.600	1.086-2.358	0.017	1.528	1.035-2.257	0.033

ER, estrogen receptor; HER2, human epidermal growth factor receptor; HSP60, Heat Shock Protein 60; PR, progesterone receptor. Bold values indicate that p < 0.05.

## Discussion

In the context of breast carcinoma tumorigenesis, HSPs are centrally involved in orchestrating cellular reactions and functions, including proliferation, invasion, and apoptosis (20, 21). HSP60 stands out as a distinct member within the HSPs family, exerting significant influence across a spectrum of biological processes within breast carcinoma (22). Desmetz et al. (23) demonstrated a statistically significant increase in Hsp60 protein levels in breast carcinoma in contrast to adjacent normal mammary tissue samples. Arya g et al. (24) observed that HSP60, when localized to the ER, induces a pro-apoptotic effect by downregulating the expression of anti-apoptotic proteins in cancer cells undergoing apoptosis triggered by the chloroform fraction of E. alba (CFEA). Furthermore, HSP60 is implicated in metastasis, with surface-bound HSP60 being linked to α3β1-integrin, a protein crucial for the adhesion of metastatic breast carcinoma cells (25). Nevertheless, there is limited understanding regarding the prognostic implications of HSP60 in breast carcinoma, and the extent to which the significance of HSP60 protein levels in breast carcinoma and their clinical relevance has been investigated is limited.

The patterns observed in this research were demonstrated by the results (1): In contrast to lower protein levels, patients with breast carcinoma and high HSP60 expression levels are expected to demonstrate a worse OS and PFS; (2) Patients with breast carcinoma exhibiting high levels of HSP60 expression are characterized by a highly aggressive clinical stage and metastatic status; (3) Compared to adjacent normal mammary tissues, HSP60 expression is elevated in tumor tissue; (4) aberrant HSP60 protein levels were not strongly related to age, pathologic stage, ER, PR, HER2 status or neoadjuvant chemotherapy in breast carcinoma patients. The findings of this research contribute insights into the outcomes of individual investigations exploring the hypothesis that HSP60 is a pivotal biomarker for breast carcinoma, thereby indicating that adjuvant therapy could be advantageous in high-risk tumor populations.

The biological mechanism underlying HSP60 also highlights its crucial involvement in breast carcinoma pathogenesis. The family of HSPs is ubiquitous across both prokaryotic and eukaryotic organisms, demonstrating evolutionary conservation. These proteins are essential for preserving cellular proteostasis and providing protection against diverse stressors (26). HSP60 has the ability to engage with p53, a gene implicated in tumor suppression, fostering cell survival in carcinoma cells by suppressing the expression of p21 and Bax. Additionally, it facilitates cancer metastasis through its interaction with β-catenin (27). Depletion of HSP60 leads to diminished levels and decreased secretion of IL-8, rendering cancer cells less tolerant to chemotherapeutic drug treatment (12). This inhibition also hinders cancer cell invasion and impedes tumor metastasis (28). The HSP60 direct contact between the apical domain and β-catenin, stimulating β-catenin activation and subsequently promoting the transcriptional activity of β-catenin and the targets it activates, laminin-γ2 and membrane-type matrix metalloproteinase-1 (MT1-MMP) (27). The association of HSP60 with β-catenin, laminin-γ2, and MT1-MMP, these entities are all markedly tied to cancer metastasis, has been firmly established (29–31). Depletion of HSP60 leads to decreased Phosphorylating and transcriptional functioning of RelA (Belonging to the NF-κB family), elevation of E-cadherin protein, and consequent suppression of tumorigenesis in cancer cells (32). Additionally, the MAPK pathway contributes to cancer initiation and progression. HSP60 is involved in mediating TPA-induced stimulation of the MAPK pathway, thereby inducing cancer cell migration (33). The absence of HSP60 leads to the inhibition of ERK phosphorylation and the progression of HCC (33), mirroring the effects observed with HSP60 downregulation in gastric carcinoma (34). Furthermore, HSP60 localized on the cell membrane can stimulate tumor migration by interacting with integrin α3β1, thus contributing to ongoing tumor metastasis (35, 36).

Our research represents the first endeavor to explore the relationships between HSP60 protein levels and clinical pathology and prognosis assessment in breast carcinoma utilizing the IHC technique. HSP60 is regarded as an oncogene, with evidence indicating that its excessive expression or pharmacological stimulation contributes to tumor advancement and is correlated with poor prognoses. Previous research has confirmed a link between increased HSP60 expression and high histological grade in breast carcinoma patients, assessed through ELISA (37). According to these findings, we propose that elevated HSP60 protein levels in breast carcinoma is indicative of an unfavorable prognosis.

Nevertheless, limitations exist in this study. Firstly, the sample size of breast carcinoma cases was rather limited. 2) the determination of HSP60 status relied solely on the detection of the protein via IHC, lacking a coordinated approach or a scoring mechanism. 3) The intricate molecular pathways involving HSP60 in breast carcinoma are still not fully understood. 4) Due to the retrospective nature and single-center design of this study, potential biases and confounding variables cannot be entirely eliminated. Additionally, lack of a uniform protocol for follow-up assessment poses a challenge. Thus, the inclusion of larger patient cohorts is imperative to advance our comprehension of HSP60’s involvement in breast carcinoma and shed light on the molecular mechanisms contributing to breast carcinoma onset.

The involvement of HSP60 in breast carcinoma treatment is significant. Studies have noted heightened levels of microRNA-29a in the serum of breast carcinoma patients. Considering HSP60’s function as a vital molecular chaperone in tumor cells, its cellular abundance can signify changes in cell behavior resulting from treatment (38). Small antisense RNA is utilized by scientists to downregulate miR-29a, leading to an increase in HSP60 levels and promoting apoptosis in breast carcinoma MCF-7 cells. The therapeutic mechanisms of this antisense RNA resemble those of Taxol. In breast carcinoma, elevating HSP60 levels can enhance tumor cell responsiveness to chemotherapy while reducing adverse reactions. This proposes that the exploration or creation of fresh chemotherapeutic medications targeting HSP60 could serve as a hopeful strategy in the management of breast carcinoma.

To conclude, our findings imply that HSP60 holds potential as a biomarker for diagnosing and prognosticating breast carcinoma, and might act as an oncogene. Additionally, gaining insights into the molecular mechanisms governing HSP60 in breast carcinoma advancement might prompt the exploration of targeted treatment strategies for breast carcinoma patients.

## Data Availability

The datasets presented in this study can be found in online repositories. The names of the repository/repositories and accession number(s) can be found in the article/**Supplementary Material**.
