# Supplementary material for: Clinicopathologic significance of heat shock protein 60 as a survival predictor in breast carcinoma
Source: Front Oncol. 2024 Aug 19;14:1415762. doi: 10.3389/fonc.2024.1415762 (PMC11366582; doi:10.3389/fonc.2024.1415762)
Supplement: Supplementary file 1 [file Table1.docx]

**S1 TABLE**｜Expression of HSP60 in breast carcinoma and para-carcinoma tissue samples.

| Variables | Overall (n) | High expression of HSP60 (n) | Low expression of HSP60 (n) | P value |
| --- | --- | --- | --- | --- |
| breast carcinoma | 42 | 30 | 12 | <0.01 |
| para-carcinoma tissue | 42 | 16 | 26 |  |
